# Reimagining Perinatal Palliative Care: A Broader Role for Support in the Face of Uncertainty

**DOI:** 10.1177/08258597221098496

**Published:** 2022-06-03

**Authors:** Sarah Lord, Rebecca Williams, Lindsay Pollard, Lori Ives-Baine, Carolyn Wilson, Kira Goodman, Adam Rapoport

**Affiliations:** 1 Division of Paediatric Medicine, Hospital for Sick Children, Toronto, Canada; 2 Paediatric Advanced Care Team, Hospital for Sick Children, Toronto, Canada; 3 Department of Paediatrics, University of Toronto, Toronto, Canada; 4 Department of Obstetrics & Gynaecology, Mount Sinai Hospital, Toronto, Canada; 5 Emily’s House Children’s Hospice, Toronto, Canada

**Keywords:** palliative care, perinatal loss, grief, ethics

## Abstract

Perinatal medicine is confronted by a growing number of complex fetal conditions that can be diagnosed prenatally. The evolution of potentially life-prolonging interventions for the baby before and after birth contributes to prognostic uncertainty. For clinicians who counsel families in these circumstances, determining which ones might benefit from early palliative care referral can be challenging. We assert that all women carrying a fetus diagnosed with a life-threatening condition for which comfort-focused care at birth is one ethically reasonable option ought to be offered palliative care support prenatally, regardless of the chosen plan of care. Early palliative care support can contribute to informed decision making, enhance psychological and grief support, and provide opportunities for care planning that includes ways to respect and honor the life of the fetus or baby, however long it may be.

## Introduction

Advances in perinatal medicine have opened doors to earlier diagnosis and intervention for fetuses with complex medical issues. For some life-threatening fetal diagnoses, medical or surgical interventions have been so successful that long-term survival and functional outcomes are undoubtedly promising.^1,2^ For others, modern science has yet to find a solution and babies will almost certainly die in utero or early in infancy.^3^ Between those two extremes fall a growing number of diagnoses for which there remains a considerable degree of prognostic uncertainty. Could a new or experimental fetal intervention improve the trajectory of a previously fatal condition? Could resuscitative efforts, high quality neonatal intensive or surgical care, and technology help prolong life long beyond expected outcomes? If a baby does survive, what will that survival look like and for how long might it be? Effectively supporting families through these challenging and often uncertain circumstances requires a flexible and multidisciplinary approach.

## Where Does Perinatal Palliative Care Fit in?

The American College of Obstetricians and Gynecologists (ACOG) defines perinatal palliative care as a strategy that “comprises options for obstetric and newborn care that include a focus on maximizing quality of life and comfort for newborns with a variety of conditions considered to be life-limiting in early infancy.”^4^ The discipline has grown over the last few decades and can include care pathways for pregnant patients experience life-threatening fetal diagnoses as well as for neonates with life-threatening conditions diagnosed after birth. In the antenatal period,^5^ perinatal palliative care continues to focus on families who have expressed a desire to: (a) continue the pregnancy, and (b) pursue a care plan focused on comfort and avoidance of intensive attempts at life-prolongation due to the severity of the fetal anomaly diagnosed prenatally.^5–11^ However, this approach only provides palliative care supports to a small subset of families facing a complex, life-threatening fetal diagnosis. ACOG also acknowledges the role of palliative care concurrently with life-prolonging treatment,^4^ and we assert that this more inclusive approach should become standard of care in perinatal practice.

Palliative care ought to be involved early following any life-threatening fetal diagnosis in order to contribute to informed decision-making and provide ongoing support through the uncertain journey, regardless of a family's decisions. Palliative care clinicians can extend additional psychosocial support to families throughout the pregnancy, as worries typically persist regardless of any post-natal choices that have been made. Even families who clearly wish to pursue life-prolonging options after delivery can benefit from ongoing support over the course of what is often a long, difficult and still uncertain journey. Depending on the circumstances, palliative care clinicians can assist with planning and providing high quality end-of-life care after birth or transition to an ongoing supportive care role for those babies that are surviving with ongoing medical complexities and mortality risk. Regardless of the plan of care, they can also help families engage in memory making and bonding activities. While the importance of these acts are openly acknowledged when a baby is nearing death,^12^ our experience has been that they can be just as important to the families we follow whose infants are receiving intensive care. Finally, palliative care teams can also provide opportunities for early and ongoing grief support in the event of the loss of the pregnancy or death of the baby.

## Ethical Considerations

When a family is faced with a life-threatening fetal diagnosis, there are a complex set of decisions to be made. The risks and benefits of pregnancy continuation versus termination may come in to question,^7^ with support needed in either case.^7,9^ The potential interventions for the fetus or newborn that may attempt to prolong life can be highly variable depending on the diagnosis. Transfer of care to different maternity care providers or facilities may also be raised as possibilities in order to access certain interventions. For parents to provide truly informed consent, it is important that they can understand and appreciate all reasonable avenues of care and what each entails. Just as a surgeon would be called in to counsel families about the potential risks and benefits of a particular surgical intervention, so too should palliative care providers be involved to describe what comfort-focused end-of-life care might look like for a baby with a particular diagnosis. Palliative care experts are well positioned to speak to the different options for symptom management as well as location of care, especially those outside of hospital (eg. home or residential hospice). Equitable access to palliative care supports should be offered to every family whose fetus has a life-threatening diagnosis where comfort-focused care may be one reasonable treatment path, regardless of whether it is the chosen path. Taking this approach ensures that the range of information and supports a given family receives is not unduly biased by the care options they pursue.

## Support Through Uncertainty

In the past, certain fetal diagnoses were considered to be “lethal”. Modern perinatal medicine acknowledges that this language is problematic, as there can be a significant degree of uncertainty in terms of prognosis when a so-called “lethal” fetal diagnosis is made.^10^ Advanced imaging and genetic testing modalities are resulting in earlier diagnoses. Even when a diagnosis is clear early on, the understanding about one baby's particular phenotype may evolve over the course of the pregnancy as its physiology, growth, and development are monitored. Technological advances in pregnancy care (eg advanced imaging technologies and fetal surgery) as well as neonatal care (surgical and intensive care supports) also continue to change. Not only is the language of “lethal” unpalatable for families, its accuracy in this context becomes questionable with continued advances in perinatal medicine.^13^

Since prognosis is often uncertain, the integration of palliative care should occur in parallel with considerations for other interventions. Palliative care focuses on exploring goals of care which can influence care planning along the trajectory. Decision making can be a fluid process as families learn and incorporate additional information from multidisciplinary providers over time.^13^ Families’ preferences and understanding of what may or may not be in their baby's best interests may change over time.^13,14^ For some, a focus exclusively on comfort or life-prolonging interventions may be clear. For others, planning for both options depending on the evolution of the pregnancy and early neonatal course best honors their goals of care.^13^ Understanding all options for how a baby will be cared for after birth also helps maternity care providers and families discuss aspects of care during pregnancy, labor, and delivery. Indeed, the goals of care that inform the plan of care for the baby may also inform labor and delivery decisions, such as the location of delivery, degree of fetal monitoring, and threshold for interventions such as caesarean section in the event of fetal distress. Palliative care teams can explore issues that provide useful input as maternal and neonatal care providers make recommendations for the labor and delivery process. Detailed birth plans that focus not only on medical details, but also contain elements of honoring the baby and making memories, can help families have some sense of control in a situation where they may feel that much of their ability to exert control has been lost.^15^

Uncertainty can also arise during the end-of-life course for a baby receiving comfort-focused care. For families who opt for this care option, earlier relationship building with a palliative care team can help elucidate where the best location of care might be if the baby lives beyond the initial hours or days after birth.^14^ While some families prefer to stay in hospital, others consider discharge to home or residential hospice. Palliative care teams tend to be most familiar with the variety of supports that can be made available outside of the hospital and in a particular community. They can also remain involved over time so that in the event that a child survives longer than expected, goals of care can continue to be revisited with familiar providers.

## Addressing Grief and Bereavement

The grief associated with carrying a baby with a life-threatening problem begins at the time of diagnosis, not at the moment the baby dies. Even for babies who have a promising future with successful medical interventions, families often grieve a change in their child's potential or loss of presumed health and a “normal” future.^16^ Palliative care supports can assist families in acknowledging grief and accessing supports early, making connections with their unborn child or early in the neonatal period through legacy building activities in order to facilitate healthier grieving later on.^9,17,18^ There are also situations where the risk of complicated grief is higher, such as for parents who experience a pregnancy termination as a result of the fetal diagnosis, parents who have no surviving children or issues with fertility, or women who do not have a supportive partner.^19^ Early intervention can also facilitate timely, appropriate referral for the small subset of patients for whom broader mental health or psychiatric support may be needed.^20^ For parents who do experience a fetal or neonatal death, the need for grief support often heightens in the acute period after the loss; however, it may also become just as relevant months or years down the road. Certain life events, such as a subsequent pregnancy, may also call for increased support.^9^ Palliative care providers can help families navigate how to access grief supports if and when they are needed in the future.^9,20^ Forming these connections early can also facilitate parents’ awareness on the anticipated grief journey over time.

## Accessing Palliative Care Supports

The ways in which perinatal palliative care providers can support primary maternal-fetal care providers and teams can look different depending on where a pregnant woman is cared for and the supports that she and the family have access to already. Many patients with a life-threatening fetal diagnosis are referred to high risk obstetrical centers where access to perinatal palliative care supports are becoming more commonplace as standalone programs or as part of the work of broader pediatric palliative care teams.^21^ Some community-based hospitals have also provided this care when families choose care options that can be delivered closer to home.^22^ For hospitals without local expertise in this area, virtual consultation and ongoing support – both for families and maternity care providers - is also possible in this context,^23^ particularly when perinatal palliative care supports are not available locally. Although in-person consultation may be ideal for some providers and families, our institutions’ experience has been that high quality perinatal palliative care can be facilitated through virtual visits and digital resources.

## Conclusions

Perinatal palliative care involvement can provide an important extra layer of support in addition to the many other providers who help patients and families facing a life-threatening fetal diagnosis. Whether or not the goals of care or trajectory of the fetal disease are fully elucidated, palliative care teams can contribute to informed decision-making, navigate uncertainty, and provide grief support. Normalizing the added benefit of palliative care early on following a fetal diagnosis can help families and palliative care teams build a longitudinal therapeutic relationship. Whether the palliative care team ultimately takes on a supportive care role for a baby receiving intensive interventions or a more central role in facilitating high quality end of life care, their support through uncertainty and grief should be seen as additive. Early perinatal palliative care involvement for all families facing a life-threatening fetal diagnosis can help optimize care throughout the pregnancy and beyond.
